# Experiential Value in Multi-Actor Service Ecosystems: Scale Development and Its Relation to Inter-Customer Helping Behavior

**DOI:** 10.3389/fpsyg.2020.593390

**Published:** 2021-01-12

**Authors:** Patrick Weretecki, Goetz Greve, Jörg Henseler

**Affiliations:** ^1^Hamburg School of Business Administration, Hamburg, Germany; ^2^Faculty of Engineering Technology, Chair of Product-Market Relations, University of Twente, Enschede, Netherlands; ^3^Nova Information Management School, Universidade Nova de Lisboa, Lisbon, Portugal

**Keywords:** customer engagement, multi-actor service ecosystem, prosocial behavior, interaction attitude, inter-customer helping behavior, scale development, experiential value

## Abstract

Interactions in service ecosystems, as opposed to the service dyad, have recently gained much attention from research. However, it is still unclear how they influence a customer’s experiential value and trigger desired prosocial behavior. The purpose of this study is to identify which elements of the multi-actor service ecosystem contribute to a customer’s experiential value and to investigate its relation to a customer’s interaction attitude and inter-customer helping behavior. The authors adopted a scale development procedure from the existing literature. Service, brand, retail and tourism management research as well as expert feedback is used to generate a pool of 33 items. Exploratory and confirmatory factor analysis (CFA) were conducted. The scale was validated based on more than 468 responses to a CASI at one of the world’s largest trade shows. Scale-development procedure was followed by structural equation modeling. CFA supports that experiential value in multi-actor ecosystems comprises five dimensions. The functional value of personnel (professionalism), the perception of other customers’ appearance (similarity), the perception of other customers’ behavior (suitable behavior), multisensory stimuli (sensory appeal), and a customer’s enjoyment (playfulness). Experiential value positively and directly relates to a customer’s interaction attitude and inter-customer helping behavior. Furthermore, the effect of experiential value on inter-customer helping behavior is partially mediated by interaction attitude. Managers interested in getting more out of interactions with customers will develop an understanding for the interplay between the physical environment and individuals within a multi-actor ecosystem. Social scientists and managers can use the scale to assess experiential value, encourage a customer’s interaction attitude and utilize the customers’ influence on their peers. This paper synthesizes insights from existing research on experiential value, from various fields, in one scale. This holistic approach is the first to simultaneously account for a customer’s interactions with the multisensory physical environment, personal interactions with employees and interactions between customers in a multi-actor service ecosystem.

## Introduction

People often act to benefit other people (e.g., helping an individual in need; sharing personal resources; volunteering time, effort, and expertise; cooperating with others to achieve some common goals), these interactions are examples of prosocial behavior (Stangor and Walinga, 2010). This behavior can be observed, in dyadic constellations, as well as in multi-actor ecosystems but also in different contexts and environments (e.g., business environment). For example, customers are increasingly interacting within service ecosystems, serving as pseudo-marketers, actively, and voluntarily contributing to marketing functions, such as customer acquisition, expansion, retention, product innovation, and marketing communication, often at lower costs and greater effectiveness than professionals (Harmeling et al., 2018; Hollebeek et al., 2018). Managers face the challenge of influencing customers in a way that they will be both motivated and empowered to contribute to the firm and engage in value co-creation behavior, as this is critical in facilitating customer engagement (Harmeling et al., 2018). The concept of customer engagement is directly connected to the evolving role of customers and the idea that they have something of value to add to the firm beyond their financial contribution (Harmeling et al., 2017). To date, research on customer engagement has predominantly focused on a customer’s interactions with specific focal objects such as the product, the firm or the frontline employees while overlooking the importance of customer-to-customer (C2C) interactions (Alexander et al., 2018). In particular, the growing importance of prosocial behaviors, like inter-customer helping behavior as a form of interaction within a service ecosystem, has become a promising research avenue (Kim, 2017; Yi and Kim, 2017). Recent research emphasizes the importance of broadening the scope of engagement research toward a holistic approach toward engagement in multi-actor service ecosystems (Harmeling et al., 2017; Li et al., 2017; Fehrer et al., 2018; Ho et al., 2020; McColl-Kennedy et al., 2020; Mustak and Plé, 2020).

“There is growing consensus that understanding prosocial behavior will require a multidimensional approach that considers the variety of [influences] that may lead to different prosocial responses” (Dunfield, 2014). One important step toward this goal is to learn more about the experiences in multi-actor service ecosystems and understand what constitutes the experiential value in these systems. Experiential events that bring different actors together in one physical space represent an ideal example of multi-actor service ecosystems (Harmeling et al., 2017). Companies worldwide have realized that experiential events can provide engaging, pleasurable, memorable and meaningful experiences to customers and thereby maximize their experiential value (Grewal et al., 2009; Brodie et al., 2011; Varshneya et al., 2017). Well-designed experiential events generate shifts in the customer value co-creation attitude, which consequently leads to customer engagement (McAlexander and Schouten, 1998; Schouten et al., 2007). Experiential events are often socially interactive experiences, meaning that multiple employees and customers share the same physical environment at the same time (Howard and Gengler, 2001). This is particularly important because a customer’s reaction to an experience can contribute to the overall experience of other customers (Grove and Fisk, 1997). Therefore, it is necessary to adopt a broader perspective that goes beyond dyadic interactions, and toward interactions among groups of actors, in multi-actor service ecosystems (Li et al., 2017; Vargo and Lusch, 2017). Thus, the design of experiential events is critical for motivating customers to engage in interaction with fellow customers, employees, and the physical environment and for the firm’s ability to motivate prosocial behavior and empower customers to share knowledge and engage in effective dialog in ways that make it impactful (Harmeling et al., 2018). However, thus far, researchers and practitioners do not know which elements of multi-actor service ecosystems (e.g., fellow customers) encourage prosocial behavior like inter-customer helping behavior. As the voluntary contributions, that customers, make, can have not only value-creating but also value-destroying outcomes, it is imperative to close this knowledge gap (Echeverri and Skålén, 2011).

Thus, the purpose of this study is to identify which elements of the multi-actor service ecosystem contribute to a customer’s experiential value and to investigate whether and how it relates to inter-customer helping behavior. Furthermore, our research examines the relevance of a customer’s interaction attitude for the relationship between experiential value and inter-customer helping behavior. This was achieved through a scale-development procedure, followed by structural equation modeling.

Our research contributes to the existing literature on quantitative psychology and measurement, as well as to literature on customer engagement marketing, in three ways. First, our study identifies the underlying experiential value dimensions of experiential events, respectively, multi-actor service ecosystems. Therefore, we provide managers and social scientists with a tool to better understand, design, and evaluate multi-actor service ecosystems. This knowledge can also be used to understand the relationship between customers and utilize customers’ influence on their peers. Second, we included existing experiential value scales, from adjacent fields, into our scale-development procedure. In this way, we are not only simply developing a new scale, but we are also contributing by highlighting how existing scales could be improved. Third, our holistic approach is the first to simultaneously account for a customer’s interactions with the multisensory physical environment, their personal interactions with employees and interactions between customers. This will be useful to managers and social scientists in understanding the interplay between the physical environment and individuals within a multi-actor ecosystem.

The remainder of this paper is structured as follows: first, the theoretical background is outlined, along with the presentation of relevant constructs (i.e., value, attitude, behavior) and their relation to one another. Moreover, this second section displays how the research hypotheses derived from knowledge gaps in the existing literature. The third section is focused on the methods used in this study and elaborates on the measures, sample, and data collection as well as the data analysis method. The fourth section summarizes the results of the data analysis. The section “Discussion” discusses the paper’s implications for researchers and practitioners before the section “Limitations and Future Research” summarizes the study’s limitations and further research implications.

## Theoretical Background

### Relationship Among Value, Attitude, and Behavior

Engagement research states that behaviors are causally driven by customer attitudes toward a firm (Kumar and Pansari, 2016; Pansari and Kumar, 2017; Petersen et al., 2018; Bergel et al., 2019). How the constructs of behavior and attitudes are related to the elements of multi-actor service ecosystems remains unclear. One possible approach to understand the interplay in multi-actor service ecosystems is through the value-attitude-behavior (VAB) model. Researchers have reported findings of the mediating role of attitudes between values and behaviors in their work and succeeded in providing an explanation (Shim and Eastlick, 1998; Jayawardhena, 2004; Hansen, 2008; Cai and Shannon, 2012; Kang et al., 2015; Shamim et al., 2017; Kautish and Sharma, 2019; Singh et al., 2019; Razali et al., 2021). According to this framework, values both directly and indirectly influence behavior (Homer and Kahle, 1988). However, the main feature of the model is its emphasis on the mediating role of attitudes between values and behaviors (Milfont et al., 2010). Values can be grouped into three underlying dimensions: internal values, external values, and interpersonal values (Homer and Kahle, 1988). Internal values are internally validated and do not require the presence of others (Kropp et al., 2005). In contrast, external values generally require the judgments or opinions of other actors (e.g., other customers within the service ecosystem) (Homer and Kahle, 1988). Interpersonal values, such as fun or enjoyment, involve an interactive motivation to fulfil the values with others (Gurel-Atay et al., 2010). Attitudes are distinct from values and best described as a predisposition to respond in a favorable or unfavorable manner with respect to a given object (Fishbein and Ajzen, 1975). Within the VAB model, behaviors represent the outcome of prior influences. Researchers have validated the principles of the VAB model for a variety of different value-attitude-behavior combinations, in different industries and contexts. For example, Shim and Eastlick (1998) researched the influence of personal values on mall shopping attitudes and behavior. Hansen (2008) investigated the influence of consumers’ personal values on their attitude and behavior regarding online grocery shopping. Kang et al. (2015) examined the influence of individual health values on individuals’ attitude and behavior regarding purchasing healthy food items. Shamim et al. (2017) provided initial evidence that the experiential value of the physical environment influences the value co-creation attitude, which subsequently influences value co-creation behavior.

### Value in Multi-Actor Service Ecosystems

Multi-actor service ecosystems are characterized primarily by interactions among groups of actors as opposed to dyadic interactions. Multiple actors (e.g., employees and customers) experience the same physical environment at the same time. Simultaneously, these actors themselves consciously or unconsciously shape and influence the experience of other actors. Values derived from experiences via interactions involving either the direct usage or indirect observation of goods or services, can be captured through experiential value (Mathwick et al., 2001). Therefore, we have focussed our attention on the experiential value of multi-actor service ecosystems. Consequently, this value must account for both external values (interaction with employees, other customers, and the physical environment) and interpersonal values (fun, enjoyment/entertainment).

The concept of experiential value, as well as the definition provided by Mathwick et al. (2001), is theoretically largely based on the work of Holbrook (1999), who defined value as an interactive, relativistic preference experience, characterizing a subject’s experience of interacting with a product or a service. Mathwick et al. (2001) proposed a four-dimensional scale comprising aesthetics, playfulness, service excellence and customer return on investment, which was tested and validated in a catalog and internet shopping context. However, while this experiential value scale is probably the most widely used, we argue that, due to its context-specificity, it is not sufficient to capture experiential value in multi-actor service ecosystems.

We propose the following revisions and extensions:

Aesthetics, due to the context of their study, are limited to visual appeal and entertainment-related factors (Mathwick et al., 2001). There is evidence that all sensory drivers (visual, acoustic, haptic, and olfactory) are significantly relevant and that all senses should be addressed within a marketing concept (Wiedmann et al., 2018). Therefore, we believe that the multisensory environment should be evaluated holistically, especially in multi-actor service ecosystems.

Service excellence, currently, reflects only the generalized consumer appreciation of a service provider (Mathwick et al., 2001). We believe that, in multi-actor service ecosystems, the functional value of the contact personnel is highly relevant for the overall experience. Therefore, the professionalism of contact personnel, as proposed by Sánchez et al. (2006), should also be considered.

Playfulness currently reflects entertainment value and escapism. Entertainment value is reflected in the intrinsic enjoyment that comes from engaging in activities, while escapism is the aspect of playfulness that allows customers to temporarily “get away from it all” (Mathwick et al., 2001). We believe that these constructs are also relevant to experiential value in multi-actor service ecosystems.

Due to its context, the scale provided by Mathwick et al. (2001) does not account for the impact of other customers on experiential value. Brocato et al. (2012) found evidence that other customers influence service experience in a retail setting and identified its relevant dimensions. These other customer perception (OCP) dimensions are “similarity” to other customers and the “physical appearance” and “suitable behavior” of other customers. We propose that the OCP dimensions should also be considered in a multi-actor service ecosystem.

### Interaction Attitude in Multi-Actor Service Ecosystems

Interaction, in multi-actor service ecosystems, takes place among multiple actors, including employees and customers, and a shared physical environment (Grönroos, 2011; Vargo and Lusch, 2016). The services cape can encourage social interaction among and between employees and customers (Bitner, 1992). Interactions help companies obtain knowledge about customer tastes and preferences (Srinivasan et al., 2002), which can be used to achieve more profitable customer relationships (Ramani and Kumar, 2008). In line with Shamim et al.’s (2017) definition, we define interaction attitude in multi-actor service ecosystems as the customer’s willingness, caused by experiential factors, to respond favorably to interaction opportunities with employees, other customers, and the physical environment.

Research shows that experiential value influences various customer attitudes. Keng and Ting (2009) found that experiential value positively influences a customer’s attitude toward engaging in blogs. Maghnati and Ling (2013) provided evidence for the positive effect of experiential value on usage attitudes toward mobile apps. Furthermore, Shamim et al. (2017) in their study found a positive direct effect of experiential value on a customer’s value co-creation attitude. Drawing on these findings, we hypothesize the following:

H_1_: Experiential value in multi-actor service ecosystems positively influences a customer’s interaction attitude.

### Inter-Customer Helping Behavior in Multi-Actor Service Ecosystems

Inter-customer helping is a prosocial behavior in form of C2C interaction. Helping refers to customer behavior seeking to assist other customers who are displaying a need for help in a service encounter (Yi and Gong, 2013). The willingness to help fellow customers is a type of customer citizenship behavior (CCB), which is voluntary and not required for the successful delivery of a service but adds additional value to the firm (Groth, 2005). Companies can benefit from utilizing inter-customer helping behavior in several ways. C2C helping enhances service value perceptions and customer loyalty intentions (Gruen et al., 2007). Inter-customer helping behavior entails a cost-saving potential through successful prevention of service failures, despite reduced employee presence (Yi and Kim, 2017). Evidently, inter-customer helping behavior is favorable, but there is a lack of knowledge in the literature about how companies can encourage this behavior.

There is some evidence that attitude can function as a major predictor of behavior. For example, Shim and Eastlick (1998) found that the attitude toward shopping malls was a direct predictor of mall shopping behavior. Hansen (2008) found that consumers’ attitude toward online grocery shopping was the most important predictor of actual buying intentions. Furthermore, attitudes, formed through direct (first-hand) experiences, are more predictive of behavior (Fazio and Zanna, 1981). We consider experience in multi-actor service ecosystems to be a suitable example of a direct experience. Therefore, this study hypothesizes the following:

H_2_: Interaction attitude in multi-actor service ecosystems positively influences inter-customer helping behavior.

Furthermore, we suspect that an experience itself influences inter-customer helping behavior. While numerous studies link values to behavior, it has also become apparent that some values relate more strongly to behaviors than others (Bardi and Schwartz, 2003). Specifically, research indicates “that stimulation and tradition values relate strongly to the behaviors that express them; hedonism, power, universalism, and self-direction values relate moderately; and security conformity, achievement, an benevolence values relate only marginally” (Bardi and Schwartz, 2003). Experiential value and inter-customer helping behavior in multi-actor service ecosystems share properties with hedonism, as they are related to pleasure and sensuous gratification for oneself (Bardi and Schwartz, 2003), but also with power and benevolence. With respect to the relationship between experiential value and inter-customer helping behavior, this study hypothesizes the following:

H_3_: Experiential value in multi-actor service ecosystems positively influences inter-customer helping behavior.

**FIGURE 1:**
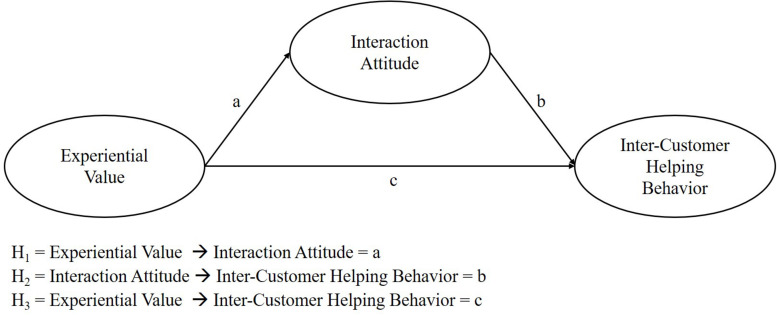
Conceptual model.

If all three proposed hypotheses can be validated, we expect that (in line with the VAB model) interaction attitude mediates the relationship between experiential value and inter-customer helping behavior. The three hypotheses are depicted in Figure 1.

## Materials and Methods

### Measures

Given the described lack of a scale to measure the experiential value in multi-actor service ecosystems, we decided that scale development and investigating the correlation has to precede testing for causation and running experiments. Experiential value in multi-actor service ecosystems neither can be observed directly nor are there existing scales to assess it. Therefore, scale development is appropriate (DeVellis, 2016). We follow Carpenter’s (2018) scale development procedure, a combination of “scale development best practices that yield stronger concepts.” Fifty-seven items for measuring experiential value in the multi-actor service environment were gathered from previous studies in the fields of service marketing, brand management, retail, and tourism management (Mathwick et al., 2001; Sánchez et al., 2006; Brocato et al., 2012; Wiedmann et al., 2018). Constructs of experiential value included sensory appeal (visual, acoustic, haptic, and olfactory), playfulness, service excellence, customer return on investment, functional value of personnel, and other customer perception. Selection and identification of relevant items and dimensions were supported by expert interviews and prior qualitative research. The wording of the items was adapted to fit the context of this research. As recommended by DeVellis (2016), we asked a panel consisting of six experiential event experts (three senior marketing executives and three project managers) of one of the biggest German companies (by revenue) and two marketing professors, to evaluate the items regarding their relevance and clarity of wording, resulting in an item pool of 33 items.

We developed measures for interaction attitude toward the physical environment, employees and other customers from the customer value co-creation attitude scale proposed by Shamim et al. (2017).

Inter-customer helping behavior measurements were extracted from the customer value co-creation behavior scale developed by Yi and Gong (2013). In their study, they demonstrated the impact of other customers on customers’ behavior in social contexts. Therefore, it is applicable to multi-actor service ecosystems.

We measured the scale with the help of a standardized questionnaire using seven-point Likert scales (1 = strongly agree, 7 = strongly disagree). Table 1 provides an overview the constructs, (sub)dimensions and measurements of experiential value, interaction attitude, and inter-customer helping behavior in multi-actor service ecosystems.

**TABLE 1:** Constructs and measurements of experiential value, interaction attitude, and inter-customer helping behavior in multi-actor service ecosystems.

**Construct**	**Dimension**	**Sub-dimension**	**Items**
**Experiential value**			
	Playfulness^a^	Escape	The experience of XYZ “gets me away from it all”
			I get so involved that I forget everything else
			The experience makes me feel like I am in another world
		Entertainment	The enthusiasm of the XYZ is catching, it picks me up
			XYZ doesn’t just sell products–it entertains me
	Service excellence^a^		When I think of XYZ, I think of excellence
			I think of XYZ as an expert in the merchandise it offers
	Customer return on investment^a^	Efficiency	Shopping from XYZ is an efficient way to manage my time
			Shopping from XYZ makes my life easier
			Shopping from XYZ fits with my schedule
	Functional value of personnel^b^	Professionalism	The personnel knew their job well
			The personnel knew their products
			The personnel’s advice was valuable
			The personnel were good professionals and they were up-to-date about new items and trends
	Sensory appeal^c^	Olfactory	The interaction area smells very nice
			The scent of the interaction area is very pleasant
			The fragrance of XYZ is very appealing
		Acoustic	The music of XYZ is very nice to listen to
			The sound scape of XYZ is very pleasant
		Haptic	The materials of XYZ feel absolutely good
			The furnishings of XYZ are very nice to touch
		Visual	XYZ is visually appealing
			The way the company displays XYZ is appealing
	Other	Similarity	The other patrons are like me
	Customer perception^d^		I could identify with the other patrons in the facility
			I liked the appearance of the other patrons
			I am similar to the other patrons in the facility
			The other patrons looked nice
		Suitable	I found that the other patrons behaved well
		Behavior	Other patrons’ behavior was appropriate for the setting
			The other patrons’ behavior was pleasant
			The other patrons were dressed appropriately
			The other patrons were friendly towards me
**Interaction attitude^e^**			
			I like to interact with elements of the environment
			I like to interact with the personnel for information seeking
			I like to interact with other customers
			I like to interact with the personnel to share information
**Inter-customer helping behavior^f^**			
			I assist other customers if they need my help
			I help other customers if they seem to have a problem
			I teach other customers to use the service correctly
			I give advice to other customers

*^a^Mathwick et al. (2001); ^b^Sánchez et al. (2006); ^c^Wiedmann et al. (2018); ^d^Brocato et al. (2012); ^e^Shamim et al. (2017); ^f^Yi and Gong (2013).*

### Sample and Data Collection

The customer survey was conducted at IFA 2018, the world’s leading experiential event for consumer electronics and home appliances. Visitors to the experiential event were intercepted and screened for appropriateness after their visit, near the exits of a 5,000 square-meter experience area. The qualifying criteria for the participants required the display of interest in the experiential offerings (exclusion of customers only passing through). Computerized self-administered questionnaires were used. Data were collected from August 31 to September 5, 2018. Out of 632 qualified respondents, 164 were discarded based on outliers and incomplete answers, resulting in 468 respondents for further analysis. Among the 468 respondents, most of the respondents were male (66.8%), in their twenties (34.3%), employed (71.5%), and had either a high school degree (41%) or a degree from a university (41.9%). Table 2 provides a detailed overview of the sample.

**TABLE 2:** Demographic profile of the sample.

**Variable**	**Characteristics**	**Frequencies**	**%**
Age	16–19 years	71	15.2
	20–29 years	160	34.3
	30–39 years	71	15.2
	40–49 years	60	12.7
	50–59 years	64	13.6
	60–65 years	24	5.1
	66 years and older	18	3.9
Gender	Female	155	33.2
	Male	313	66.8
Education	High School	192	41.0
	University	196	41.9
	Without higher education	80	17.1
Occupation	Full time/part time	335	71.5
	Unemployed	133	28.5
Occupation (if unemployed)	Pupil	39	29.5
	Student	46	34.5
	Pensioner	34	25.9
	House wife/husband	5	3.6
	Other	9	6.5

*N = 468.*

## Data Analysis

To achieve the purpose of this study and test the hypotheses, SPSS and AMOS statistics package programs were used. Exploratory factor analysis (EFA) using SPSS 25.0 was performed to discover the number of factors of the experiential value scale for experiential events. Using AMOS 25.0, confirmatory factor analysis (CFA) was conducted to examine the validity of the scale, while structural equation modeling was used to test the hypotheses. Convergent validity was assessed based on the average value extracted (AVE), with a recommended cut-off of 0.5 (Hair et al., 2010). Discriminant validity was evaluated by checking whether the AVE of each construct was greater than the inter-construct correlations (Hair et al., 2010). Composite Reliability (CR) was used to evaluate internal consistency, with a threshold of 0.7 for the CR values. The reliability for each construct was assessed based on Cronbach’s α.

## Results

### Dimensions of Experiential Value in Multi-Actor Service Ecosystems

Prior to investigating the relationship between experiential value and a customer’s interaction attitude, the dimensions of experiential value in multi-actor service ecosystems (EX-MAS) had to be identified. The sample was checked for adequacy. The results show a value of KMO = 0.955, which is considered excellent for factor analysis, and MSA values for all items above 0.70, indicating a high degree of inter-correlation among items (Kaiser and Rice, 1974). Bartlett’s test of sphericity indicates a chi-square value of 14,891.187 with 1,035 degrees of freedom and a *p* = 0.000 < 0.05, confirming that the correlation matrix is not an identity matrix.

To identify the underlying factor structure and reduce the number of items to the optimum, a series of principal axis factoring with oblique rotation (oblimin) was iteratively performed. In an oblique rotation, the supposition is that there is correlation among two or more of the factors rotated (Pett et al., 2003). Prior research has provided evidence that the constructs, considered by us, are in fact correlated (Brocato et al., 2012; Mathwick and Mosteller, 2017; Shamim et al., 2017). We conducted common factor analysis instead of principal component analysis because the results are considered more generalizable when submitting hypothesized models to a CFA (Haig, 2005; Worthington and Whittaker, 2006; Carpenter, 2018).

Social scientists, concerned about the optimal number of factors, should determine them based on theory and multiple tools (Carpenter, 2018). Hence, we used multiple methods and validated with prior research on similar topics. In our case, the five-factor structure was deemed the best because it explains a considerable amount (60.03%) of the total variance, the eigenvalue greater than one rule was followed and the screen test (visual plot of eigenvalues) clearly supported that structure as well as prior research. The results of the principal axis factoring after the purification process (deletion of five items) are shown in Table 3. Skewness and kurtosis indices were within an acceptable range of −2 and +2 (George and Mallery, 2018). Means ranged from 1.53 to 3.55 and standard deviations from 0.79 to 1.73. An examination of the items comprising each factor led to naming them as follows: professionalism, similarity, suitable behavior, sensory appeal, and playfulness.

**TABLE 3:** Results of principal axis factoring.

**Rotated factor matrix**	**Factor 1^a^**	**Factor 2^b^**	**Factor 3^c^**	**Factor 4^d^**	**Factor 5^e^**
The personnel knew their job well	0.829				
The personnel knew their products	0.755				
The personnel’s advice was valuable	0.746				
The personnel were good professionals and they were up-to-date about new items and trends	0.741				
The other patrons are like me		0.705			
I could identify with the other patrons in the facility		0.651			
I liked the appearance of the other patrons		0.636			
I am similar to the other patrons in the facility		0.633			
The other patrons looked nice		0.559			
I found that the other patrons behaved well			0.681		
Other patrons’ behavior was appropriate for the setting			0.666		
The other patrons’ behavior was pleasant			0.630		
The other patrons were dressed appropriately			0.518		
The other patrons were friendly toward me			0.476		
The interaction area smells very nice				0.919	
The scent of the interaction area is very pleasant				0.879	
The fragrance of XYZ is very appealing				0.847	
The music of XYZ is very nice to listen to				0.626	
The sound scape of XYZ is very pleasant				0.601	
The materials of XYZ feel absolutely good				0.565	
XYZ is visually appealing				0.471	
The furnishings of XYZ are very nice to touch				0.443	
The way the company displays XYZ is appealing				0.332	
The experience of XYZ “gets me away from it all”					0.809
I get so involved that I forget everything else					0.772
The XYZ makes me feel like I am in another world					0.738
The enthusiasm of the XYZ is catching, it picks me up					0.676
XYZ doesn’t just sell products—it entertains me					0.470

*^a^Professionalism; ^b^Similarity; ^c^Suitable Behavior; ^d^Sensory Appeal; ^e^Playfulness; Extraction method: Principal axis factoring; Rotation method: Oblimin rotation.*

Subsequently, CFA was performed on the items identified in the exploratory factor analysis to analyze its model fit, reliability, and validity (Anderson and Gerbing, 1988). The five-factor structure was confirmed. However, a closer investigation of the model indicated that both sensory appeal and playfulness have underlying sub-constructs, and are therefore best designed as second-order constructs (Khadraoui and Gharbi, 2012). The model fit indices Comparative Fit Index (CFI), Tucker-Lewis Index (TLI), Normed Fit Index (NFI), and Root mean square error of approximation (RMSEA) represented a good fit with CFI (0.955), TLI (0.949), NFI (0.921), CMIN/DF (2.232), RMSEA (0.052), and PCLOSE (0.275). All items loaded significantly on their respective factors, and the AVE for all five factors was acceptable (> 0.50), indicating convergent validity (Hair et al., 2010). The composite reliabilities for all five factors exceeded the recommended level of 0.70 and therefore were acceptable (i.e., professionalism = 0.880; similarity = 0.871; playfulness = 0.943; suitable behavior = 0.894; sensory appeal = 0.930). As shown in Table 4, the proposed model achieved discriminant validity, as the square root of the AVE for all three factors, depicted on the diagonal, was greater than the inter-construct correlations (Hair et al., 2010). Thus, the scale was deemed reliable and valid.

**TABLE 4:** Test of composite reliability, convergent, and discriminant validity.

**Dimension**	**CR**	**AVE**	**Professionalism**	**Similarity**	**Playfulness**	**Suitable behavior**	**Sensory appeal**
Professionalism	0.880	0.648	**0.805**				
Similarity	0.871	0.575	0.469**	**0.758**			
Playfulness behavior	0.943	0.893	0.589**	0.585**	**0.956**		
Suitable behavior	0.894	0.629	0.613**	0.754**	0.501***	**0.793**	
Sensory appeal	0.930	0.768	0.764**	0.541**	0.706***	0.643***	**0.876**

**p < 0.01, ***p < 0.001. CR = Composite reliability; AVE = Average Variance Extracted; Square root of AVEs (on the diagonal in bold).

The first construct, professionalism, relates to perceived knowledge, valuableness of information and competence of employees within the service ecosystem. The factor loadings of the items ranged from 0.78 to 0.83, with a Cronbach’s α of 0.88. The second construct, similarity, refers to the extent to which customers feel that they are similar to and can identify with other customers. The factor loadings of the items ranged from 0.72 to 0.84, with a Cronbach’s α of 0.88. Construct 3, suitable behavior, may be interpreted as the extent to which a customer feels that the other customers in the multi-actor service ecosystem behave appropriately. The factor loadings of the items ranged from 0.74 to 0.83, with a Cronbach’s α of 0.89. The fourth construct, sensory appeal, relates to the influence of the multisensory environment on the experiential value. Sensory appeal has four sub-constructs (olfactory, acoustic, haptic, and visual). The factor loadings for the sub-constructs ranged from 0.80 to 0.95, with a Cronbach’s α of 0.93. The last construct, playfulness, represents the influence of experienced entertainment and temporarily escape from the daily routine. Factor loadings for the sub-constructs ranged from 0.86 to 1 with a Cronbach’s α of 0.88. A sensitivity analysis was conducted to check for potential threats to the estimates’ validity. As suggested by Eggert et al. (2012), we modeled incrementally increasing measurement item error correlations. The analysis revealed that even if the error correlations were one third of the loadings, our substantial conclusions would remain unaffected. Table 5 summarizes the findings of the CFA.

**TABLE 5:** Confirmatory factor analysis (CFA) for experiential value in multi-actor service ecosystems.

**Construct 1st order**	**2nd order**	**Items**	**Factor loading**	**Cronbach’s α**
Professionalism		The personnel knew their job well	0.78	0.88
		The personnel knew their products	0.83	
		The personnel’s advice was valuable	0.78	
		The personnel were good professionals and they were up-to-date about new items and trends	0.83	
Similarity		The other patrons are like me	0.73	0.88
		I could identify with the other patrons in the facility	0.84	
		I liked the appearance of the other patrons	0.76	
		I am similar to the other patrons in the facility	0.72	
		The other patrons looked nice	0.73	
Suitable behavior		I found that the other patrons behaved well	0.74	0.89
		Other patrons’ behavior was appropriate for the setting	0.82	
		The other patrons’ behavior was pleasant	0.82	
		The other patrons were dressed appropriately	0.83	
		The other patrons were friendly toward me	0.75	
Sensory appeal	(Olfactory)	The interaction area smells very nice	0.80 (0.91)	0.93 (0.93)
		The scent of the interaction area is very pleasant	(0.90)	
		The fragrance of XYZ is very appealing	(.92)	
	(Acoustic)	The music of XYZ is very nice to listen to	0.81 (0.85)	(0.85)
		The sound scape of XYZ is very pleasant	(0.88)	
	(Haptic)	The materials of XYZ feel absolutely good	0.95 (0.87)	(0.85)
		The furnishings of XYZ are very nice to touch	(0.85)	
	(Visual)	XYZ is visually appealing	0.93 (0.83)	(0.77)
		The way the company displays XYZ is appealing	(0.77)	
Playfulness	(Escape)	The experience of XYZ “gets me away from it all”	0.86 (0.81)	0.88 (0.84)
		I get so involved that I forget everything else	(0.81)	
		The XYZ makes me feel like I am in another world	(0.77)	
	(Entertainment)	The enthusiasm of the XYZ is catching, it picks me up	1.0 (0.88)	(0.77)
		XYZ doesn’t just sell products–it entertains me	(0.74)	

*N = 468; χ^2^ = 741; df = 332; p = 0.001; NFI = 0.92, TLI = 0.95, CFI = 0.95, RMSEA = 0.05.*

The results indicate that experiential value in multi-actor service ecosystems is in fact highly influenced by other customers and employees. Therefore, their impact cannot be neglected.

### Inter Customer Helping and Attitude

Additionally, we conducted a CFA of the constructs interaction attitude and inter-customer helping behavior. The latent constructs were correlated, whereas the measurement items and their error items were constrained to be uncorrelated. The CFA achieved good fit (SRMR = 0.076, NFI = 0.96, IFI = 0.97, CFI = 0.97, RMSEA = 0.08, CMIN/DF = 4.077).

### Structural Model

The structural model was tested. The overall model fit was satisfactory. The model resulted in a chi-square statistic (χ^2^ = 1,448, *df* = 580) and acceptable fit indices (SRMR = 0.055, NFI = 0.88, IFI = 0.93, CFI = 0.92, RMSEA = 0.05). The factor loadings of all constructs were significant, with an AVE greater than 0.50 providing evidence for convergent validity. As shown in Table 6, the square roots of all three constructs represented in the diagonal are greater than the inter-construct correlations, indicating discriminant validity. The proposed modifications to the Mathwick et al. (2001) scale revealed an increase of 48% of observable experiential value. A direct comparison between this new model and the one provided by Mathwick et al. (2001) indicated that the model fit of the new scale is considerable better. In comparison to the new model, the Mathwick et al. (2001) model only achieved a chi-square statistic (χ^2^ = 1,175, *df* = 270) and the fit indices (SRMR = 0.074, NFI = 0.82, IFI = 0.86, CFI = 0.86, RMSEA = 0.09).

**TABLE 6:** Validity matrix (complete model).

**Dimension**	**CR**	**AVE**	**Interaction attitude**	**Inter-customer helping behavior**	**Experiential value**
Interaction attitude	0.801	0.513	**0.716**		
Inter-customer helping behavior	0.881	0.649	0.359***	**0.806**	
Experiential value	0.891	0.620	0.655***	0.419***	**0.787**

***p < 0.001. CR, Composite reliability; AVE, Average Variance Extracted; Square root of AVEs (on the diagonal in bold).

**FIGURE 2:**
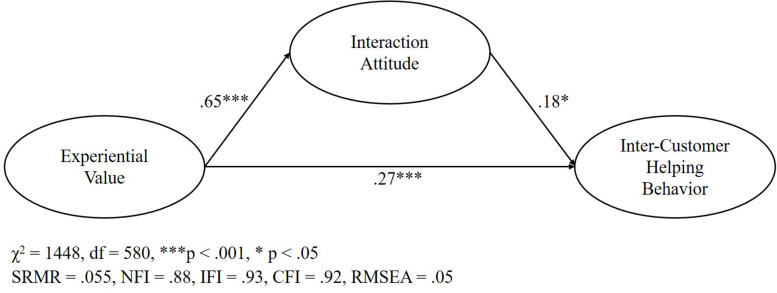
Results of the structural equation model.

After the overall model fit was approved, hypotheses were tested via structural equation modeling. The structural equation model’s standardized path coefficients were used to evaluate the hypotheses (see Figure 2).

H_1_ predicts that experiential value in multi-actor service ecosystems positively influences a customer’s interaction attitude. As presented in Table 7, the hypothesis is strongly supported. The standardized path coefficient between experiential value and interaction attitude is β = 0.65, CR = 12.08 and *p* < 0.001.

**TABLE 7:** Hypothesis testing results.

**Hypothesis**	**Effect**	**Path coefficient**	**SE**	**CR**	***p***	**Remarks**
H1	Experiential value → Interaction attitude	0.65	0.066	12.08	0.001	Supported
H2	Interaction attitude → Inter-customer helping behavior	0.18	0.137	2.480	0.013	Supported
H3	Experiential value → Inter-customer helping behavior	0.27	0.166	3.637	0.001	Supported

*SE, Standard error; CR, Composite reliability.*

The second hypothesis (H_2_) suggests that interaction attitude in multi-actor service ecosystems positively influences inter-customer helping behavior. For this path, with a standardized path coefficient of β = 0.18, CR = 2.480 and *p* < 0.05, the hypothesis was also supported.

The third hypothesis (H_3_) suggests that experiential value in multi-actor service ecosystems positively influences inter-customer helping behavior. For this path, with a standardized path coefficient of β = 0.27, CR = 3.637 and *p* < 0.001, the hypothesis was also supported.

Considering that both H_1_ and H_2_ have been supported, point in the same direction and are significant, we suspect that interaction attitude mediates the relationship between experiential value and inter-customer helping. The indirect effect of experiential value on inter-customer helping behavior must be significant to establish the mediation effect. We used the state-of-the art approach for mediation analysis as suggested by Zhao et al. (2010) and performed a bootstrapping procedure with 2,000 bootstrap samples and used the 90% bias-corrected confidence level. The results of the analysis revealed a significant indirect effect of experiential value on inter-customer helping behavior via interaction attitude [β = 0.012, *p* = 0.048, standard deviation (SD) = 3.02, 95% CI (0.05, 0.52)], supporting partial mediation of interaction attitude. The mediated effect (a × b) and the direct effect (c) (β = 0.273, *p* = 0.001, *SD* = 3.46) point in the same direction, indicating complementary mediation (Zhao et al., 2010). The total effect, direct effect and the indirect effect are presented in Table 8.

**TABLE 8:** Effects of experiential value on inter-customer helping behavior.

**Effect**	**Coefficient**	**Error probability**
Total effect (ab+c)	0.285	0.001
Direct effect (c)	0.273	0.001
Indirect effect (ab)	0.012	0.048
Conclusion: Partial mediation (complementary mediation)		

## Discussion

The aim of this study was to identify which elements of the multi-actor service ecosystem contribute to a customer’s experiential value and to investigate its relation to a customer’s interaction attitude and inter-customer helping behavior. To achieve this objective, we applied a scale development procedure and explored the underlying experiential value dimensions in a multi-actor ecosystem. Thereafter, a structural model was tested.

This study implied certain cause-and-effect relationships between the independent and dependent variables. However, we are aware that, in order to establish causality, we would need three conditions fulfilled: Concomitant variation between the supposed independent and the supposed dependent variable, temporal precedence of the independent variable, as well as exclusion of other plausible alternative explanations. In the proposed model, the standardized path coefficients (of H_1_, H_2_, and H_3_) are equal to the correlation. As evidenced by their magnitude, the first condition (concomitant variation) can be assumed. Simultaneously, this correlation is clear evidence of the external validity of the experiential value scale. The scale is evidence for the, formerly ignored, relevance of interactions between customers for the experiential value. However, if that is the case, it is not implausible to assume a reversed causal effect, i.e., that inter-customer helping behavior may also affect experiential value (in the sense that helping others creates value in itself). Hence, temporal precedence is still strongly suspected but cannot be asserted with absolute certainty. As already pointed out, we decided that given the described lack of a scale, scale development and investigating the correlation has to precede testing for causation and running experiments. This choice of research design limits our ability to fully exclude other plausible alternative explanations at this point, as the best method for this would be conducting a carefully designed experiment (Moore et al., 2012). In summary, our results indicate a high degree of external validity and a valid new scale. The findings of our study support the following conclusions:

First, experiential value in multi-actor ecosystems comprises five dimensions. It is based on the functional value of personnel (professionalism), the perception of other customers’ appearance (similarity), the perception of other customers’ behavior (suitable behavior), multisensory stimuli (sensory appeal), and a customer’s enjoyment (playfulness). Our findings highlight the importance of adopting a holistic approach toward experiential value in multi-actor service ecosystems. The personal interaction between customers and employees and interactions among customers in multi-actor service ecosystems account for three out of five relevant experiential value dimensions. Managers may use this finding and invest in proper coaching and training activities for employees with direct customer contact, who might not have the necessary skills and knowledge required, nor be aware of their importance. This is particularly important because the appropriate level of social interaction (i.e., interaction intensity, frequency, etc.) largely depends on the type of service and must fit to a customer’s expectation. Our findings also suggest that perceptions of other customers’ appearance and behavior impact a customer’s experiential value. Managers need to be aware of and accept the fact that there are drivers of experiential value outside of their direct sphere of influence. One possible way to counteract the risks associated with other customers’ appearance and behavior is by attempting to limit the appeal of an offering (e.g., product, service) or an event (e.g., experiential event, promotion, campaign) to a preferred customer clientele. Obviously, this comes at a cost that managers must consider. For example, advertising and promoting a sweepstakes is a common method to increase in-store customer frequency. However, this entails the risk that “sweepstakes hunters,” who are very different in appearance and behavior from the “usual clientele,” might negatively influence the experiential value of other customers present in the ecosystem. Furthermore, our analysis confirmed that multi-actor service ecosystems are in fact multisensory and that the sensory appeal is formed by olfactory, acoustic, haptic, and visual stimuli. This is not an entirely new finding. However, while there are companies that have achieved substantial competitive advantage via the creation of a multisensory customer experience, some of the best-publicized multisensory store designs have been extremely expensive (Spence et al., 2014). Managers need to be aware of these facts and consciously weigh increased experiential value, through optimized multisensory appeal, against high investments with uncertain return on investment. Furthermore, our analysis points to the importance of playfulness for the EX-MAS. Designing an experience such that it entertains and functions as a temporarily escape from the daily routine might prove challenging for two reasons. First, it might be in conflict with a company’s objective to communicate information (e.g., product/service specifications, price). Managers must weigh providing a fun and exciting experience against their own goals with regard to the messages they want to convey. Second, depending on the product/service, customers might expect different levels of playfulness. This is challenging because, in most cases, multiple products/services are displayed simultaneously. Because the possibility to stage products/services in a certain way is limited, due to limited budgets or availability of physical space, managers probably have to prioritize some over others. Researchers can greatly benefit from these findings, as the developed scale can be used to measure experiential value more accurately than before, especially, in cases of multi-actor ecosystems.

Second, our analysis supported the hypotheses that experiential value relates to a customer’s interaction attitude and inter-customer helping behavior. The direct relation on interaction attitude is relevant for several reasons. For example, managers can use this information to increase the number of interactions between customers and employees. This adds potential for customer acquisition but also the potential to increase revenue from existing customers (e.g., cross-selling, up-selling). Furthermore, companies can utilize a customer’s direct feedback for product improvement and new product development. Customers, on the other hand, might benefit from interaction with employees through an increase in knowledge about current and future offerings (e.g., products, services, deals), which potentially increases customer satisfaction. Knowledge about the positive influence of experiential value on inter-customer helping behavior is also highly relevant to companies. Managers considering a reduction of service personnel can attempt to utilize customers as “pseudo employees” to compensate for such downsizing. However, professionalism is also a part of experiential value; therefore, managers must identify the optimal ratio of employees in relation to pseudo employees.

Third, our analysis supported the positive effect of interaction attitude on inter-customer helping behavior, and the mediating effect of interaction attitude between experiential value and inter-customer helping behavior. This strengthens the importance of correctly identifying and influencing experiential value because it shows a significant direct and indirect relation to inter-customer helping behavior.

## Limitations and Future Research

This study is the first to make an effort to identify experiential value in multi-actor service ecosystems. Unlike previous studies, we simultaneously considered a customer’s interaction with the multisensory physical environment, as well as personal interaction with employees and between customers, as sources of experiential value. The 28 items of the proposed scale demonstrate that experiential value is based on the functional value of personnel (professionalism), perception of other customers’ appearance (similarity), perception of other customers’ behavior (suitable behavior), multisensory stimuli (sensory appeal), and visitors’ enjoyment (playfulness). Our study provides empirical validation for some of the findings of other researchers (e.g., Mathwick et al., 2001; Sánchez et al., 2006; Varshneya and Das, 2017; Wiedmann et al., 2018). While it succeeds in answering the question on which elements of multi-actor service ecosystems contribute to a customer’s experiential value, and in investigating its relation to a customer’s interaction attitude and inter-customer helping behavior, due to our research setting, we had the fortune (or misfortune) of a “competitor-free environment.” However, in multi-actor service ecosystems without full constructional separation (e.g., shop-in-shop), there might be additional influences on a customer’s experiential value (e.g., employees of the competition, multisensory stimuli). Researchers and practitioners are invited to validate the EX-MAS scale in those cases where other companies noticeably compete for a customer’s attention.

The goal of this study was to contribute similarly to theory and practice by developing an experiential value scale for multi-actor service ecosystems and investigating the correlations between experiential value, interaction attitude, and inter-customer helping behavior. This study used correlational and (customers’) self-reported data. Going forward, we suggest an experimental design examining the hypothesized relationships to support causal conclusions. Additionally, future research can try to observe actual customer behavior and compare it to the self-reported data from this study. It is necessary to validate the scale and investigate its applicability across different countries, cultures and industries.

Furthermore, our research employed a cross-sectional survey design. Although our study established substantial correlation between experiential value, interaction attitude and inter-customer helping behavior, we encourage future research to explore the longitudinal effects. Specifically, it could be interesting to investigate whether inter-customer attitude needs to be reinforced once “generated” and, if so, at which intervals and which intensity. In addition to that, future research should address and control for potential endogeneity.

We identified a complementary mediation in our proposed model. The significant direct effect of experiential value on inter-customer helping behavior “points to the possible existence of some omitted second mediator that can be pursued in future research” (Zhao et al., 2010). Future research is invited to continue with the investigation of additional mediators. For example, it may prove insightful to take a closer look at individuals perceived responsibility. There is some evidence that perceived responsibility mediates the relationship between an experience in the presence of others (multi-actor service ecosystem) and inter-customer helping behavior (Yi and Kim, 2017).

This study focused on experiential value and on evaluating its impact on a customer’s interaction attitude and inter-customer helping behavior. Future researchers may continue with the investigation and contribute to the literature by studying if experiential value can also be used to foster other prosocial behaviors, dimensions of the customer citizenship behavior (e.g., feedback, advocacy, tolerance) or customer participation behavior and how that can be done both effectively and efficiently.

## Data Availability Statement

The datasets presented in this article are not readily available because individual approval is necessary. Requests to access the datasets should be directed to PW.

## Ethics Statement

Ethical review and approval was not required for the study on human participants in accordance with the local legislation and institutional requirements. Written informed consent to participate in this study was provided by the participants, and where necessary, the participants’ legal guardian/next of kin.

## Author Contributions

PW was responsible for the conceptualization, the literature review, the methodology, the investigation, and for drafting the document. GG contributed to the formal analysis, review, and editing of the document. JH contributed to the positioning, the theorizing, and the methodology and supervised the research process. All authors contributed to the article and approved the submitted version.

## Conflict of Interest

The authors declare that the research was conducted in the absence of any commercial or financial relationships that could be construed as a potential conflict of interest.
